# Supplementary material for: Analysis of PPARγ Signaling Activity in Psoriasis
Source: Int J Mol Sci. 2021 Aug 10;22(16):8603. doi: 10.3390/ijms22168603 (PMC8395241; doi:10.3390/ijms22168603)
Supplement: Supplementary file 1 [file ijms-22-08603-s001.zip › Supplemental materials_Analysis of PPARg signaling activity in psoriasis/Pathway models/Models images and html files/PPARG inhibits phenotypes assotiated with psoriasis/143117.html]

degranulation


# Cell Process degranulation

|  |  |
| --- | --- |
| URN | urn:agi-cellproc:degranulation |
| Total Entities | 3 |
| Connectivity | 2544 |
| Name | degranulation |

---

|  |  |
| --- | --- |
| ChildConcepts | platelet exocytosis |
|  | leukocyte degranulation |
|  | mast cell degranulation |

---

|  |  |
| --- | --- |
| Pathway | Basophil Activation |
|  | Eosinophil Activation |
|  | Mast-Cell Activation via IgE Signaling |
|  | Mast-Cells De Novo Synthesized Mediators via IgE Independent Signaling |
|  | Neutrophil Recruitment and Priming |
|  | Neutrophil Activation via FCGR3B |
|  | Neutrophil Degranulation via FPR1 Signaling |
|  | Neutrophil Chemotaxis |
|  | Neutrophil Activation via Adherence on Endothelial Cells |
|  | SNS Related Adrenal Cortex Response |
|  | Eosinophil Activation and Degranulation in Asthma |
|  | Neutrophil Degranulation via FPR1/IL8 |
|  | Basophil Activation in Asthma |
|  | Mast-Cell Activation in Asthma |
|  | PPARG inhibits phenotypes assotiated with psoriasis |
|  | efects selected |
|  | 2\_1\_Eosinophilia and neutrophilia in asthma: eosinophil migration and activation |
|  | 2\_2\_Eosinophilia and neutrophilia in asthma: eosinophil apoptosis |

---

|  |  |
| --- | --- |
| MedScan ID | 4003445 |

---

|  |  |
| --- | --- |
| Alias | cell degranulation |
|  | granulocyte degranulation |
|  | degranulation |
|  | degranulations |
|  | degranulatio |
|  | de-granulation |
|  | cell degranulations |

---
